# Cafestol Inhibits High-Glucose-Induced Cardiac Fibrosis in Cardiac Fibroblasts and Type 1-Like Diabetic Rats

**DOI:** 10.1155/2020/4503747

**Published:** 2020-12-31

**Authors:** Ju-Chi Liu, Po-Yuan Chen, Wen-Rui Hao, Yi-Chung Liu, Ping-Chiang Lyu, Hong-Jye Hong

**Affiliations:** ^1^Division of Cardiology, Department of Internal Medicine, Shuang Ho Hospital, Taipei Medical University, New Taipei, Taiwan; ^2^Department of Biological Science and Technology, College of Biopharmaceutical and Food Sciences, China Medical University, Taichung, Taiwan; ^3^Institute of Population Sciences, National Health Research Institutes, Miaoli County, Taiwan; ^4^Institute of Bioinformatics and Structural Biology, National Tsing-Hua University, Hsinchu, Taiwan; ^5^School of Chinese Medicine, College of Chinese Medicine, China Medical University, Taichung, Taiwan

## Abstract

Diabetes is associated with the development of myocardial fibrosis, which is related to various cardiac diseases. Cafestol, one of the active ingredients in coffee, has been reported to exert biological effects. However, whether cafestol can ameliorate diabetes-induced cardiac fibrosis remains unknown. The aim of this study was to evaluate the effects of cafestol on cardiac fibrosis in high-glucose-treated cardiac fibroblasts and streptozocin- (STZ-) induced diabetic rats. Rat cardiac fibroblasts were cultured in high-glucose (25 mM) media in the absence or presence of cafestol, and the changes in collagen synthesis, transforming growth factor-*β*1 (TGF-*β*1) production, and related signaling molecules were assessed on the basis of ^3^H-proline incorporation, enzyme-linked immunosorbent assay, and western blotting. Cardiac fibroblasts exposed to high-glucose conditions exhibited increased collagen synthesis, TGF-*β*1 production, and Smad2/3 phosphorylation, and these effects were mitigated by cafestol treatment. Furthermore, cafestol increased the translocation of nuclear factor erythroid 2-related factor 2 and increased the expression of heme oxygenase-1. The results of molecular docking analysis suggested a selective interaction of cafestol with Kelch-like ECH-associated protein 1. The rats with untreated STZ-induced diabetes exhibited considerable collagen accumulation, which was ameliorated by cafestol. Moreover, activities of catalase, superoxide dismutase, general matrix metalloproteinase, and reduced glutathione concentration were upregulated, whereas malondialdehyde level was downregulated by treatment with cafestol in rats with cardiac fibrosis. These findings highlight the effects of cafestol, which may be useful in treating diabetes-related cardiac fibrosis.

## 1. Introduction

Diabetes mellitus is a leading cause of cardiovascular diseases which influence the myocardium in several aspects. Among them, cardiac fibrosis is one of the major pathological processes that contribute to the deterioration of cardiac function during diabetic cardiomyopathy [1]. In cardiac fibrosis, the surfeit production of the extracellular matrix proteins can change the structure and function of the heart and matrix metalloproteinases (MMP) play a crucial role in facilitating alteration and remodeling. Although a number of mediators are involved in this process, the most important is hyperglycemia which causes glycation of proteins and triggers reactive oxygen species (ROS) production [1]. Besides, numerous mechanisms have been proposed regarding the development of myocardial fibrosis in diabetes. Increasing evidence has demonstrated that transforming growth factor-*β*1 (TGF-*β*1), a prosclerotic cytokine, is involved in cardiac fibrosis and that its expression is increased in patients with diabetes [2]. Moreover, high-glucose concentrations (or hyperglycemia) have been demonstrated to stimulate cardiac fibroblast proliferation, increase fibroblast collagen production, and mediate increases in TGF-*β*1 activity and downstream canonical Smad signaling [3]. Furthermore, cardiac fibrosis was demonstrated to be significantly induced by the administration of streptozotocin (STZ) in a diabetic animal model [4]. These studies have indicated that oxidative stress plays a critical role in the diabetic induction of myocardial fibrosis. Therefore, increasing the antioxidant capacity of cardiac tissue may be an efficient approach to preventing or postponing the development of cardiac fibrosis in patients with diabetes. However, effective and safe approaches to prevent diabetic induction of myocardial fibrosis for diabetic patients are not currently available.

Cafestol, one of the active ingredients of the berries of *Coffea arabica* L. (Rubiaceae) [5], reportedly exhibits insulinotropic effects on pancreatic *β*-cells, stimulates glucose uptake in human skeletal muscle cells [6], and attenuates hyperglycemia in mice with diabetes [7]. Nuclear factor erythroid-2-related factor 2 (Nrf2) is a transcription factor that regulates basal and inducible transcription of genes encoded with protective molecules against various forms of oxidative stress [8]. Activated Nrf2 mediates the induction of antioxidant proteins, such as heme oxygenase-1 (HO-1), through the antioxidant response element-dependent pathway [8]. Triggering Nrf2 antioxidant signaling could protect against several cardiovascular diseases (hypertension, cardiac hypertrophy, and cardiomyopathies) and provide cardioprotection against myocardial ischemia and reperfusion injury [9]. Moreover, cafestol has been documented to activate Nrf2/HO-1, thereby inhibiting urotensin II- (U-II-) induced interleukin-8 expression and cell proliferation in endothelial cells [10] and preventing U-II-induced cardiomyocyte hypertrophy [11]. Cafestol treatment may represent a new strategy for treating oxidative stress-related pathophysiological damage. However, the effects of cafestol on diabetes-induced cardiac fibrosis have not been directly addressed. In this study, we investigated the effect of cafestol on hyperglycemia-induced cardiac fibrosis in both rats and cultured cardiac fibroblasts. Furthermore, we explored mechanisms associated with cafestol-induced antifibrotic signals in the diabetic heart.

## 2. Material and Methods

### 2.1. Chemicals

Cafestol acetate (purity ≥98%) and all other reagent-grade chemicals were purchased from the Millipore Sigma (St. Louis, MO, USA). Fetal calf serum, Dulbecco's modified Eagle's medium (DMEM), and tissue culture reagents were purchased from Thermo Fisher Scientific (Grand Island, NY, USA). The primary antibodies anti-Nrf2, anti-HO-1, and Keap-1 were purchased from Santa Cruz Biotechnology (Santa Cruz, CA, USA). The primary antibodies anti-p-Smad2/3, anti-Smad2/3, anti-GAPDH, and anti-Poly (ADP-ribose) polymerase (PARP) were purchased from Cell Signaling Technology (Boston, MA, USA).

### 2.2. Cardiac Fibroblast Cell Culture and Cell Proliferation Assay

Primary cultures of neonatal rat cardiac fibroblasts were prepared as described in a previous study [10]. In brief, ventricles from 1- to 2-day-old neonatal Sprague-Dawley rats were minced and subjected to 0.125% trypsin. Pooled cell suspensions were centrifuged and resuspended in DMEM supplemented with 10% fetal calf serum. Cardiac fibroblasts were isolated by removal of myocytes through selective adhesion of nonmyocytes at a 2 h preplating interval. Nonmyocytes attached to the bottom of the dishes were subsequently incubated for an additional 2–4 days. Confluent cardiac fibroblasts from the second to fourth passage were used in the experiments. After incubation with cafestol for 12 h, cardiac fibroblasts were exposed to a serum-free normal glucose medium (5.6 mM glucose) or a high-glucose medium (25 mM glucose) for an additional 24 h before analyses. Proliferation was assessed by quantifying 5-Bromo-2′-deoxyuridine (BrdU) incorporation as described in a previous study [10].

### 2.3. 3H-Proline Incorporation Assay

A high-glucose medium (25 mM) with or without cafestol was used for the stimulation experiments, and the incorporation of exogenous ^3^H-proline was evaluated by scintillation counting as described in a previous study [10].

### 2.4. Measurement of TGF-*β*1 Concentrations

TGF-*β*1 concentrations were measured in a culture medium by using a commercial enzyme-linked immunosorbent assay (ELISA) kit (R&D Systems Inc., Minneapolis, MN, USA) in accordance with the manufacturer's instructions [10].

### 2.5. Western Blot Analysis

Cytoplasmic and nuclei components of cardiac fibroblasts were isolated using a nuclei isolation kit (NUC201, Sigma-Aldrich) in accordance with the manufacturer's instructions. Western blot analysis was performed as described in a previous study [10]. The proteins were visualized using chemiluminescence in accordance with the manufacturer's instructions (Pierce ECL Western Blotting Substrate, Thermo Fisher Scientific). The PARP detection was used as a loading control for nuclear fractions. Quantification was performed by densitometry. The results were normalized to GAPDH or PARP. All experiments were performed three times.

### 2.6. Immunofluorescence Staining

Cardiac fibroblasts were grown on sterile glass coverslips in 6-well plates and pretreated with cafestol or with a vehicle (dimethyl sulfoxide, DMSO) for 6 h before being exposed to the high-glucose medium for 6 h. The coverslips were then incubated with the Nrf2 antibody (diluted 1 : 200) and washed with phosphate buffered saline prior to incubation with an anti-rabbit-FITC antibody (diluted 1 : 200). Immunofluorescence images were captured using a fluorescence microscope (Eclipse; Nikon, Tokyo, Japan) equipped with a digital camera (DXM1200; Nikon) [11].

### 2.7. Molecular Docking Analysis

Molecular docking was performed to investigate the binding mode of cafestol to human Kelch-like ECH-associated protein-1 (Keap1). The crystal structure of the Kelch domain of human Keap1 (PDB ID: 1U6D) was used to screen cafestol by molecular docking with RyRx/AutoDock VINA (ver. 0.9.7) [12]. The three-dimensional (3D) structure of cafestol was obtained from the National Center for Biotechnology Information (PubChem CID: 108052). A grid box with the maximum size for this 3D model was generated using AutoGrid embedded in PyRx/AutoDock Vina. The docking results were analyzed according to the predicted binding mode and corresponding binding affinity (kcal/mol). Docked complexes were visualized and analyzed using the PyMOL Molecular Graphics System (Ver. 2.0 Schrödinger, LLC) and LigPlot^+^ 2.1 program (https://www.ebi.ac.uk/thornton-srv/software/LigPlus/) [13].

### 2.8. Coimmunoprecipitation Analysis

Cells pretreated with a proteasome inhibitor MG132 (10 *μ*M) for 2 h were lysed at 4°C in lysis buffer [11]. The cell lysates were incubated at 30°C for 2 h in the presence or absence of cafestol (30 *μ*M) followed by using immunoprecipitation kits (Thermo Fisher Scientific) with anti-Nrf2 antibody and protein-G-agarose according to manufacturer's instructions. The precipitates were washed with a lysate buffer, and the Nrf2 and KEAP1 proteins in the immunocomplex were monitored by western blot analysis.

### 2.9. Diabetic Rat Model, Experimental Design, and Masson's Trichrome Staining

The investigation conformed to the US National Institutes of Health's Guide for the Care and Use of Laboratory Animals (Publication No. 85-23, revised 1996), and all animal experimental procedures were approved by the Institutional Animal Care and Use Committee of Taipei Medical University (IACUC 2016-032). Male Sprague-Dawley rats (*n* = 20, weight, 220–260 g) were housed in a controlled environment (12 h light/dark cycle, 25 ± 2°C and 50% ± 60% humidity) with ad libitum access to standard rat chow and autoclaved water throughout the studies. Research was conducted over a 6-week period. Single intraperitoneal injections of STZ (60 mg/kg body weight) were employed to induce diabetes in fasting rats, as described in another study [14]. The rats were randomly assigned to four groups, comprising the normal control group (*n* = 5, administered a vehicle orally daily for 6 weeks and an intraperitoneal injection of sodium citrate buffer vehicle at day 15), cafestol group (*n* = 5, administered cafestol orally at a dose of 1 mg/kg body weight daily for 6 weeks and a sodium citrate buffer vehicle through intraperitoneal injection at day 15), STZ group (*n* = 5, administered a vehicle orally daily for 6 weeks and STZ at a dose of 60 mg/kg body weight through intraperitoneal injection at day 15), and cafestol plus STZ group (*n* = 5, administered cafestol orally at a dose of 1 mg/kg body weight daily for 6 weeks and STZ at a dose of 60 mg/kg body weight through intraperitoneal injection at day 15). Before and after STZ injection, blood glucose was monitored every week. Blood glucose measurements were collected after 4 h of fasting. Blood was obtained through puncture of the tail vein, and blood glucose levels were measured using a glucometer (Accu-Chek; Roche, Basel, Switzerland). On the last day of the study, the rats were euthanized in a gas chamber through exposure to excess carbon dioxide. The abdomen and thorax were opened, and the whole heart was isolated, rinsed with an ice-cold saline solution, placed in 10% formalin, and embedded in paraffin. Myocardial fibrosis was detected by preparing 5 *μ*m thick sections stained using a Trichrome Stain (Masson) Kit (Sigma-Aldrich) in accordance with the manufacturer's instructions. The stained slices were observed and imaged at 200× magnification by using a phase-contrast microscope (Nikon, Tokyo, Japan). The myocardial collagen concentration was quantified using Image-Pro Plus (Media Cybernetics, Rockville, MD, USA). The collagen volume fraction is equal to the ratio of collagen area to the sum of the myocardial and collagen areas, and the mean value represents the collagen volume fraction of the section.

### 2.10. Measurement of Cardiac Antioxidant Enzyme and General MMP Activities

Heart tissues were homogenized in 10 mL phosphate buffer (50 mM, pH 7.4) and centrifuged at 15,000×g for 30 min at 4°C. A supernatant was used in accordance with the manufacturer's instructions to measure malondialdehyde (MDA) level (Lipid Peroxidation Assay Kit, Abcam, Cambridge, MA, USA), reduced glutathione (GSH) concentration (GSH/GSSG Ratio Detection Assay Kit, Abcam), catalase activity (Catalase Assay Kit, Sigma-Aldrich), superoxidase dismutase (SOD) activity (SOD Assay Kit, Sigma-Aldrich), and general MMP activity (MMP Activity Assay Kit, Abcam).

### 2.11. Statistical Analysis

Data are presented as the mean ± standard error of the mean (SEM). Statistical analyses were performed using Prism version 3.0 for Windows (GraphPad Software). Differences between groups were evaluated using unpaired *t*-tests. For multiple comparisons, one-way analyses of variance were performed and further analysis was conducted using the post hoc Tukey's test. A *P* value <0.05 was considered statistically significant.

## 3. Results

### 3.1. Cafestol Inhibits High-Glucose-Induced Cell Proliferation, Collagen Synthesis, TGF-*β*1 Secretion, and Smad2/3 Phosphorylation in Cardiac Fibroblasts

**Figure 1:**
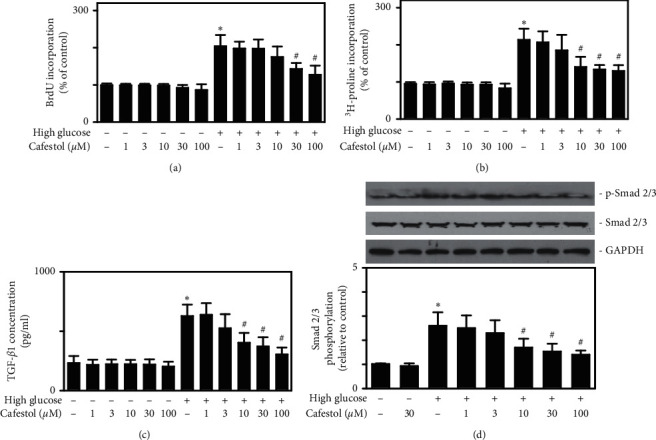
Effects of cafestol on high-glucose-induced cell proliferation, collagen synthesis, TGF-*β*1 secretion, and Smad2/3 phosphorylation in cardiac fibroblasts. Cardiac fibroblasts were cultured in a serum-free normal glucose medium (5.6 mM glucose) or high-glucose medium (25 mM glucose) in the absence or presence of cafestol (1, 3, 10, 30, or 100 *μ*M) for 24 h. (a) Cell proliferation was assessed using BrdU incorporation. The results are reported as the mean ± SEM (*n* = 5). (b) Collagen synthesis was assessed using ^3^H-proline incorporation. The results are reported as the mean ± SEM (*n* = 5). (c) The secretion level of TGF-*β*1 in cardiac fibroblasts supernatant was measured using an ELISA assay. The results are reported as the mean ± SEM (*n* = 6). (d) The protein expression levels of Smad2/3 and P-Smad2/3 were detected using western blotting. Cardiac fibroblasts were treated with a normal glucose medium or high-glucose medium in the absence or presence of cafestol (30 *μ*M) for 2 h. Representative micrograph of the expression of Smad2/3 and P-Smad2/3 in a western blot analysis (upper) and the quantitative results (lower). The results are reported as the mean ± SEM (*n* = 3). *∗P* < 0.05 versus normal glucose control group; # *P* < 0.05 versus high-glucose group.

High-glucose stress was induced in vitro by replacing the cell culture medium with 25 mM glucose. We conducted a pilot study to determine the effects of osmotic pressure on cardiac fibroblasts. The experimental groups were the normal control group (medium with 5.6 mM glucose), high-glucose treatment group (medium with 25 mM glucose), and high osmolarity group (medium with 5.6 mmol/L glucose + 19.4 mM mannitol). Cardiac fibroblasts treated for 24 h with a high-glucose medium exhibited a significant increase in cell proliferation and collagen synthesis, assessed using BrdU and ^3^H-proline incorporation (data not shown). Consistent with previous reports [15], these effects were not observed in high-osmolarity-treated cardiac fibroblasts. Therefore, these results were not due to changes in osmotic pressure. We then tested the effect of cafestol on cardiac fibroblast proliferation and collagen synthesis. Isolated cardiac fibroblast cells were cultured in a normal or high-glucose medium. Pretreatment of cardiac fibroblasts with cafestol (30 or 100 *µ*M) for 12 h followed by exposure to high levels of glucose resulted in significant reductions in high-glucose-increased cell proliferation and collagen synthesis as determined by BrdU incorporation and ^3^H-proline incorporation (Figures 1(a) and 1(b), respectively). Cytokine TGF-*β*1 and Smad signaling were demonstrated to affect the remodeling process and play a crucial role in the pathogenesis of cardiac fibrosis [2, 3]. We further examined the effect of cafestol on TGF-*β*1 secretion and Smad2/3 phosphorylation. As illustrated in Figure 1(c), cardiac fibroblasts treated with high-glucose medium exhibited increased secretion of TGF-*β*1, as determined by ELISA, compared with cardiac fibroblasts in the control group (5.6 mM glucose). However, high-glucose-induced TGF-*β*1 secretion was prevented by treatment with cafestol (10–100 *μ*M). As illustrated in Figure 1(d), high-glucose medium treatment for 2 h enhanced the phosphorylation level of Smad2/3. However, high-glucose-stimulated Smad2/3 phosphorylation was suppressed by cafestol treatment (10–100 *μ*M). These findings indicate that cafestol may inhibit high-glucose-induced proliferation and collagen synthesis by regulating the TGF-*β*1/Smad pathway.

### 3.2. Cafestol Modulates the Nrf2/HO-1 Signaling Pathway

**Figure 2:**
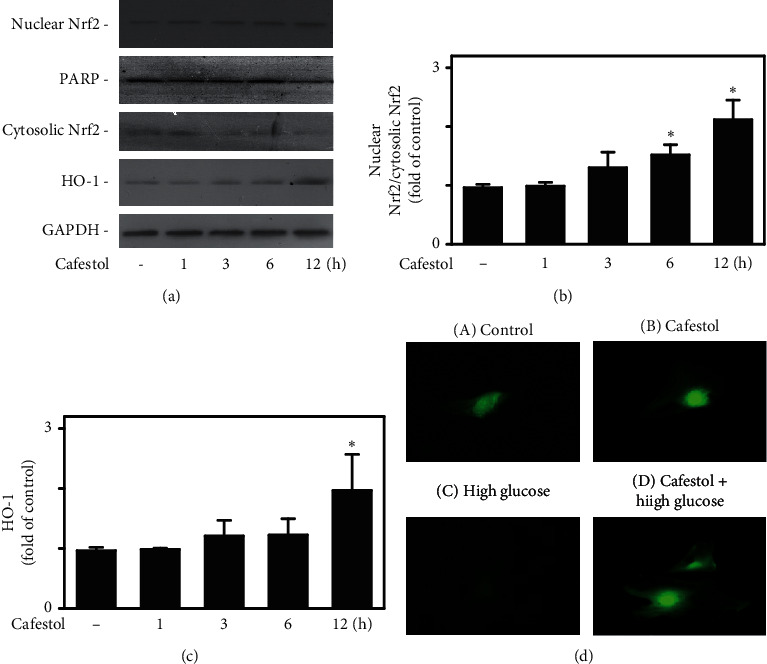
Cafestol modulated the Nrf2/HO-1 signaling pathway. (a) The effects of cafestol on Nrf2 and HO-1 expression were observed using western blotting. Cells were treated with 30 *μ*M cafestol for the time indicated. Nuclear extracts were prepared, and western blotting was subsequently performed using anti-Nrf2 and anti-HO-1 antibodies. GAPDH and PARP were used as internal controls. (b) The ratio of nuclear Nrf2/cytosolic Nrf2 is presented in the bar graphs. (c) The relative protein expression of HO-1 to GAPDH is presented in the bar graphs. Data are expressed as the mean ± SEM (*n* = 3). *∗P* < 0.05 compared with the control. (d) Cafestol enhanced Nrf2 nuclear translocation. Cardiac fibroblasts were grown on coverslips and treated with or without cafestol (30 *µ*M, 12 h before undergoing high-glucose treatment for 6 h Panels are as follows: (A) control, (B) cafestol treatment, (C) high-glucose treatment, and (D) cafestol treatment and then high-glucose treatment. After treatment, cells were fixed and permeabilized, and Nrf2 subcellular localization was determined and visualized using a fluorescent microscope (taken at 200× magnification). Images representative of a typical experiment are presented.

Cafestol has been demonstrated to inhibit U-II-induced interleukin-8 expression and cell proliferation in endothelial cells [10] and prevent U-II-induced cardiomyocyte hypertrophy [11] through Nrf2/HO-1 activation. To reveal the possible signaling pathways and mechanisms of the effects of cafestol under high-glucose conditions, we detected the translocation of Nrf2 and Nrf2 downstream of HO-1 in cardiac fibroblasts (Figure 2(a)). Our data indicated that cafestol (30 *µ*M, 12 h) treatment could induce increases in the translocation of Nrf2 from the cytoplasm to the nucleus (Figure 2(b)) and HO-1 protein expression (Figure 2(c)). To assess the effect of cafestol in Nrf2 nuclear translocation, cardiac fibroblasts were exposed to a high-glucose medium and cafestol, and the subcellular location of endogenous Nrf2 was examined using immunofluorescence with an Nrf2 antibody (Figure 2(d)). Cardiac fibroblasts were grown on coverslips and treated with or without cafestol (30 *µ*M, 12 h) before undergoing high-glucose treatment for 6 h. After the treatment, cells were fixed and permeabilized, and Nrf2 subcellular localization was determined and visualized using a fluorescent microscope. Fluorescence was fuzzy in both the nucleus and cytoplasm of untreated control cells, whereas cell exposure to cafestol, nuclear fluorescence, and cytoplasmic staining was evident. However, even though cytoplasmic fluorescence was reduced, a significant increase in nuclear fluorescence was not detected in cells undergoing high-glucose treatment. Pretreatment of cells with cafestol prior to high-glucose treatment prevented the reduction in cytoplasmic fluorescence observed under the use of high-glucose treatment alone. These data suggest a role of the Nrf2/HO-1 signaling pathway in the antifibrotic effects of cafestol.

### 3.3. Molecular Docking of Cafestol with Keap1

**Figure 3:**
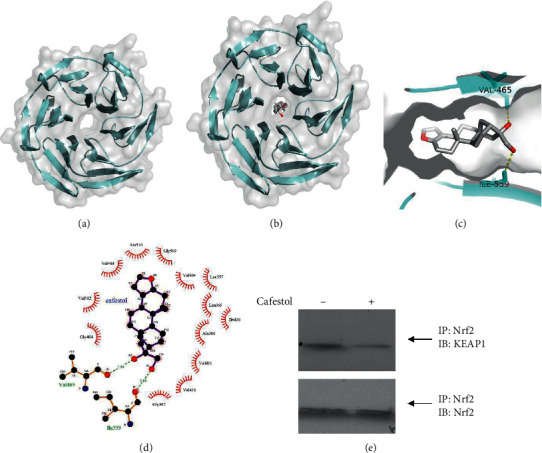
Molecular docking of cafestol with Keap1. (a) The structure of the receptor protein Keap1 (PDB: 1U6D). (b) Representative binding modes of the cafestol molecule in the active pocket of Keap1. (c) The pocket reveals that cafestol inserted into the cavity of Keap1 and the hydroxyl group of cafestol formed two hydrogen bonds with the backbone Val465 and Ile559. (d) Hydrophobic interactions and hydrogen bonding between cafestol and Keap1. LigPlot^+^ was used for visualization of protein-ligand interactions. Hydrogen bonds are illustrated as green dashed lines, and hydrophobic interactions are illustrated as red arcs. (e) Cafestol binds to KEAP1 to release Nrf2. Cardiac fibroblasts pretreated with MG132 (10 *μ*M) for 2 h then lysed with lysis buffer. The cell lysates were incubated at 30°C for 2 h in the presence or absence of cafestol (30 *μ*M) followed by using immunoprecipitation kits (Thermo Fisher Scientific) with anti-Nrf2 antibody. The Nrf2 and KEAP1 proteins in the immunocomplex were monitored by western blot analysis. Representative western blot of Nrf2 and Keap-1 protein expression (*n* = 3). IP: immunoprecipitation. IB: immunoblotting.

Keap1 is documented to repress Nrf2 activation [16]. Nrf2 is anchored in the cytoplasm by binding to Keap1, which facilitates the ubiquitination and subsequent proteolysis of Nrf2. Therefore, Keap1 is an attractive drug target [17]. The interactions of cafestol and Keap1 were studied in silico. To explore how cafestol interacts with human Keap1 (Figure 3(a)), procured from the Protein Data Bank (PDB ID: 1U6D), molecular docking analysis was performed with RyRx/AutoDock Vina to evaluate potential binding sites. The potential binding model was proposed by the program and is displayed in Figure 3(b). This model predicted that cafestol inserts into the cavity of Keap1 (Figure 3(c)), where it is stabilized by Keap1 residues through hydrophobic and hydrogen bond interactions. In this model, the furan ring of cafestol is inserted into the cavity and interacts with Val463, Gly509, and Ala510 through hydrophobic interactions (Figure 3(d)). Moreover, the hydroxyl group of cafestol interacts with the Gly367, Val418, Ile559, and Val606 through hydrophobic interactions and forms two hydrogen bonds with the backbone Val465 and Ile559. Therefore, these interactions were proposed to assist cafestol in anchoring in the Keap1 binding site. Furthermore, we evaluated whether cafestol treatment resulted in dissociation of the Nrf2-KEAP1 complex. In cell lysates treated with cafestol, Nrf2 and KEAP1 coimmunoprecipitated as determined by western blot analysis. As shown in Figure 3(e), cafestol decreased coprecipitation of the Nrf2-KEAP1 complex. KEAP1 coimmunoprecipitation with Nrf2 was markedly diminished following treatment with cafestol, indicating that cafestol treatment resulted in dissociation of Nrf2 from the Nrf2-KEAP1 complex. These results suggested that cafestol activated Nrf2 via binding to KEAP1.

### 3.4. Cafestol Attenuates STZ-Induced Myocardial Fibrosis

**Figure 4:**
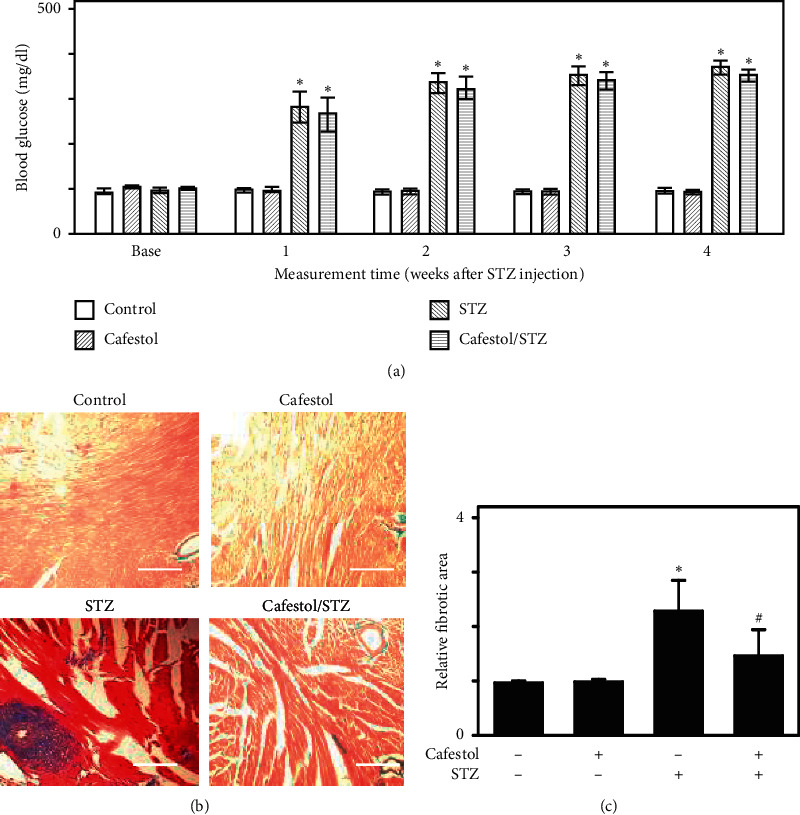
(a) Effects of cafestol on blood glucose levels in rats from the normal control group (control), the cafestol-pretreated group (cafestol), the STZ-injected group (STZ), and the cafestol-pretreated STZ-injected group (cafestol/STZ). Before and after the injection of STZ, the rats blood glucose levels were monitored weekly. (b) Effects of cafestol on cardiac fibrosis. Photomicrographs of the myocardium in the control, cafestol, STZ, and cafestol/STZ groups were characterized through staining with Masson's trichrome (Masson Stain). The scale bar is 100 *μ*m. (c) Quantitative analysis of cardiac fibrosis; the results are expressed as the mean ± SEM (*n* = 5). *∗P* < 0.05 vs the control group and # *P* < 0.05 vs the STZ group.

**Figure 5:**
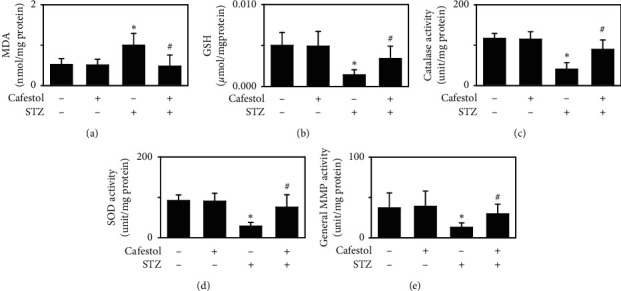
Effects of cafestol on cardiac antioxidant enzyme and general MMP activities in rat hearts in hyperglycemic states. The MDA level, GSH concentration, and activity of intracellular enzymes, such as catalase, SOD, and general MMP, were detected in the myocardium of the control group, the cafestol-pretreated group, the STZ-injected group, and the cafestol-pretreated/STZ-injected group. (a) Cardiac MDA level, (b) GSH concentration, and the activities of (c) catalase, (d) SOD, and (e) general MMP were assayed using the corresponding quantification kits. The results are expressed as the mean ± SEM (*n* = 5) *∗P* < 0.05 vs the control group and # *P* < 0.05 vs the STZ-injected group.

The effects of cafestol on hyperglycemia-induced cardiac fibrosis were further examined using an STZ-induced hyperglycemic rat model. Blood glucose levels were measured in rats from four groups (control, cafestol, STZ, and cafestol/STZ). Blood glucose concentration was significantly increased at the end of the first week and 4 weeks after STZ injection in the STZ group compared with the control group. The STZ and cafestol/STZ groups had significantly higher blood glucose levels compared with the other two groups, suggesting the successful induction of hyperglycemia. No significant differences in blood glucose levels were observed between the STZ and the cafestol/STZ groups (Figure 4(a)). To investigate the effect of cafestol on hyperglycemia-induced cardiac fibrosis, the myocardial collagen concentration was analyzed using Masson's trichrome staining (Figure 4(b)). Rats in the STZ group had significant collagen accumulation, which was primarily detected in interstitial tissues and also in the perivascular area. Cafestol treatment attenuated hyperglycemia-induced cardiac fibrosis (Figure 4(c)). We subsequently evaluated antioxidant enzyme and general MMP activities in cardiac tissues (Figure 5). The MDA level, membrane lipid oxidation products, GSH concentration, intracellular antioxidant enzyme activity, such as catalase and SOD, and total MMP activity were detected. The MDA level increased and the GSH concentration was reduced in the STZ group, which was reversed by cafestol treatment. Moreover, biological cardiac catalase, SOD, and MMP activity were lower in the STZ group and higher in the cafestol/STZ group. These results suggest that cafestol may stimulate the production of antioxidants to attenuate hyperglycemia-induced cardiac fibrosis.

## 4. Discussion

Cardiac fibrosis is related to various cardiac diseases; it is one of the major pathological processes in diabetes and manifests as hyperglycemia (high-glucose) [1]. The development of an appropriate strategy for the prevention of diabetes-induced cardiac fibrosis is crucial to stopping or postponing the development of cardiac complications. We analyzed the function of cafestol, an active ingredient in coffee beans, in preventing cardiac fibrosis using cultured cardiac fibroblasts in a high-glucose medium and an STZ-injected rat model. In the present study, we revealed that cafestol treatment may diminish both high-glucose-induced collagen synthesis and STZ-induced progression of cardiac fibrosis, which coincide with the increased translocation of Nrf2 and cellular antioxidant activity.

Studies have reported that high-glucose concentrations induce fibroblast proliferation [3], increase the deposition of structural extracellular matrix proteins [18], and activate canonical TGF-*β*1/Smad fibrogenic signals [18]. ROS have been reported to play a role in the pathogenesis of cardiac fibrosis in diabetes [19]. We determined that cafestol may act as an inhibitor of high-glucose-induced fibroblast proliferation, collagen synthesis, expression of TGF-*β*1, and Smad2/3 phosphorylation. Our previous study demonstrated that cafestol treatment can reduce the production of ROS in cultured cardiomyocytes [11]. Cafestol may have modulated high-glucose-induced cardiac fibroblast proliferation and collagen synthesis through antioxidation by inhibiting oxidative stress. Similarly, in a diabetic mice study, the results obtained from proteomics and miRNA profile in the renal cortex point out Nrf2 regulation as a potential factor involved in diabetic kidney [20]. However, our study also demonstrated that cafestol promoted Nrf2 translocation to the nucleus, which may have upregulated HO-1. Consistent with our previous studies, cafestol regulated Nrf2 activation and HO-1 expression in vascular endothelial cells and cardiomyocytes [10, 11]. Nrf2 is retained within the cytoplasm by binding with Keap1 and targeted for ubiquitin-mediated proteasomal degradation under homeostatic conditions [21]. However, under oxidative stress, Nrf2 is dissociated from Keap1 after ROS generation [22] and translocated into the nucleus to prompt target genes such as HO-1, catalase, superoxide dismutase (SOD), and glutathione to maintain ROS production [23]. In addition, Nrf2 has also been reported to inhibit the TGF-*β*1/Smad pathway, thereby attenuating dystrophic muscle fibrosis [24]. Therefore, cafestol may reduce high-glucose-induced cell proliferation and collagen synthesis through the direct antifibrotic effects of Nrf2 and indirect downregulation of ROS levels. Moreover, modification of Keap1 cysteine residues as a result of oxidative or electrophilic stress also inhibits proteasomal degradation of Nrf2 [21]. Although other potential interactive receptors and the exact induction mechanism of cafestol's activation of Nrf2 in cardiac fibroblasts remain to be determined, some inferences can be made on the basis of structural considerations. Furan epoxides and corresponding dicarbonyl derivatives have been long associated with the cellular effects of furan-containing compounds because of their high reactivity and tendency to react with oxygen and nitrogen nucleophiles as well as thiolates [11]. Thus, cafestol's furan ring may be converted into a thiol-reactive species through epoxidation and thus form adducts with biological enzymes or proteins [25]. We identified a molecular docking model based on human Keap1, which further indicated that cafestol may be Keap1 selective. This may be partially due to the conformational structure of cafestol, which leads to a stronger interaction with Keap1. The binding sites of the active center of human Keap1 have not been fully explored [26], whereas it has also been documented that polar amino acids, including Val465 and Ile559, are in the agonist-binding site in the ligand-binding domain of human Keap1 [26]. As demonstrated in our docking results, the furan ring of cafestol was positioned deep in the hydrophobic pocket of Keap1 and surrounded by Leu365, Ala366, Ile416, Val463, Val464, Gly509, Ala510, Val512, Leu557, and Val604 (Figure 3). Crucially, the hydroxyl group of the cafestol forms two hydrogen bonds with backbone Val465 and Ile559 to stabilize the binding pose in addition to hydrophobic interaction with Gly367, Val418, Ile559, and Val606. Finally, cafestol decreased co-precipitation of the Nrf2-KEAP1 complex. This protein-ligand binding mode may also provide clues for further understanding the mechanism of action of cafestol on the cardiovascular system. However, a precise understanding of its molecular binding requires additional structural studies to reveal the underlying molecular mechanism of Nrf2 activation by cafestol.

Diabetic complications are primarily related to hyperglycemia [27]. In our study, plasma glucose levels were substantially higher in the STZ-injected rats than that in the control rats, indicating the success of diabetes model induction. Moreover, we did not observe any significant difference in blood glucose levels between the STZ and cafestol/STZ groups, which is inconsistent with previous reports that cafestol reduced hyperglycemia in a KKAy mice [7]. This discrepancy may be attributed to the difference in experimental models; the previous study was performed in a KKAy mouse experimental model, whereas an STZ-injected rat model was used in the present study. Diabetes is associated with increased formation of free radicals and a reduction in antioxidant potential, which induces cardiac fibrosis [28]. In our study, STZ injection in rats also facilitated the progression of cardiac fibrosis. Furthermore, Masson's staining revealed less fibrotic deposition in the hearts of the cafestol-pretreated STZ-injected rats than in the hearts of vehicle-pretreated STZ-injected rats. Moreover, the increased oxidative stress in STZ-injected rat hearts, indicated by elevated MDA levels, was suppressed by cafestol. Similarly, STZ administration reduced GSH concentration and the enzymatic activity of catalase, SOD, and general MMP. Cafestol increased GSH concentration and potentiated the enzymatic activity of catalase, SOD, and MMP. Therefore, cafestol attenuated hyperglycemia-induced cardiac fibrosis, which may have been mediated by a mechanism involving increased antioxidant activity. However, more detailed research is needed to address this causal relationship.

Moderate coffee consumption was reported to possess antioxidant and anti-diabetic properties in diabetic state [29]. Thus, the daily intake of the coffee may improve the glycemic status and increases the antioxidant level which results in protection of the cardiac tissue damage. The present study highlighted the protective effect of the cafestol on diabetic cardiac fibrosis and encourages future research to use cafestol as a supplement to treat cardiac complications arising due to diabetes mellitus. As a result, further detailed studies are needed to explore the underlying mechanism and further clinical study is required in order to elucidate the efficacy and safety of cafestol.

## 5. Conclusions

**Figure 6:**
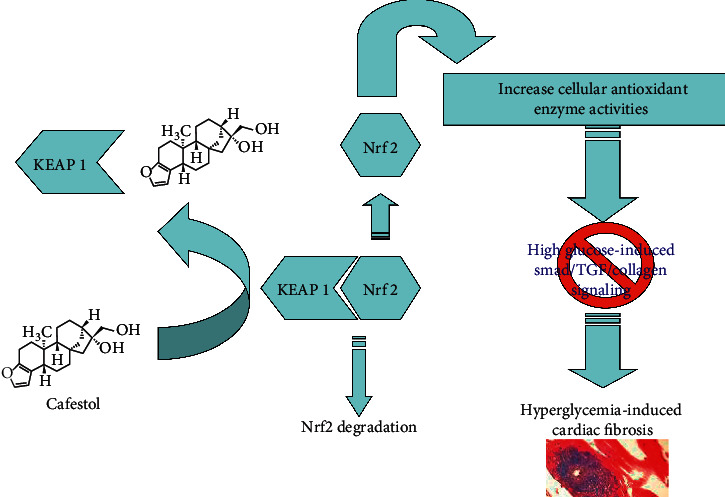
Schematic representation for the regulation of antioxidant genes by NRF2-KEAP1 interaction via cafestol under hyperglycemic condition in rat heart. Antioxidant gene expression could repress Smad/TGF/collagen signaling under hyperglycemic state. Administration of cafestol could diminish high glucose-induced fibrosis in rat heart.

Our results demonstrate that cafestol reduced high-glucose-induced collagen synthesis (as summarized in Figure 6). The antifibrotic effects of cafestol appeared to be at least partially caused by a reduction in the expression of the profibrogenic cytokine TGF-*β*1 and inhibition of Smad2/3 signaling. Moreover, pretreatment with cafestol attenuated cardiac fibrosis in STZ-induced diabetic rats. These results suggest that cafestol may have potential in preventing and treating diabetes-induced cardiac fibrosis.

## Data Availability

All data are available from the corresponding author upon reasonable request.
